# The Bio Economic Seaweed Model (BESeM) for modelling tropical seaweed cultivation – experimentation and modelling

**DOI:** 10.1007/s10811-022-02799-8

**Published:** 2022-08-19

**Authors:** P. A. J. van Oort, N. Rukminasari, G. Latama, A. Verhagen, A.K. van der Werf

**Affiliations:** 1 Wageningen Plant Research, Wageningen, The Netherlands; 2 Faculty of Marine Science and Fisheries, Hasanuddin University, Makassar, Indonesia

**Keywords:** Seaweed, Rhodophyta, *Gracilaria*, Model, Farming

## Abstract

The Bio Economic Seaweed Model (BESeM) is a model designed for modelling tropical seaweed cultivation. BESeM can simulate the common tropical seaweed cultivation system with multiple harvests per year, clonal reproduction and labour intensive harvesting and replanting activities. Biomass growth is modelled as a sigmoid, with growth being initially exponentially and eventually flattening off towards a maximum weight per plant or per square meter (*w*_*f,max*_). To estimate the latter, longer duration experiments than normal are needed – in the order of 100 days rather than 45 days. Drying (on platforms on the beach) is simulated as well as increase in harvested chemical concentration over time since planting, for harvested chemicals such as agar extracted from *Gracilaria* or carrageenan extracted from *Kappaphycus* or *Euchema*. BESeM has a limited number of parameters which makes it easily amenable to new sites and species. An experiment is presented for a site in Indonesia in which *Gracilaria* was monitored for 120 days in 6 nearby sites and from which BESeM model parameters were estimated. A simulation example is presented which illustrates how BESeM can be used to find the optimum combination of replanting weight and harvest cycle length (in days) for maximising gross and net farm income.

**Supplementary Information:**

The online version contains supplementary material available at 10.1007/s10811-022-02799-8.

## Introduction

Seaweed farming is an important source of income for coastal communities in a number of tropical countries such as India, Indonesia and the Phillipines (FAO 2018). Seaweed species *Gracilaria*, *Kappaphycus alverezii* (Cottonii) and *Eucheuma denticulatum* (Spinosum) are amongst the most commonly commercially cultivated tropical seaweeds (Santelices and Doty 1989; Dawes et al. 1994; McHugh 2003; Hurtado et al. 2014; Periyasamy et al. 2019). Tropical seaweed farming is a labour intensive process. The dominant cultivation system is one where seaweed is planted and harvested multiple times within a year, with growing periods of around 45-60 days per cycle (Valderrama et al. 2013, 2015). In most cases, part of the harvest is set aside for replanting, so seaweed reproduction is mostly clonal (as opposed to the cultivation system where inoculated lines are bought from specialised companies). At sea shores around the equator such as in Indonesia, marine environmental conditions are fairly constant, except for rough weather during the monsoon season. Cultivation along the shore is possible for at 6-12 months, depending mainly on roughness of the sea.

Two important farm management decisions are (1) when to harvest and (2) how much to replant. As long as the seaweed continues growing, yield per harvest will be higher with a longer cycle. A farmer with shorter harvest cycles (e.g. 30 instead of 60 days) can have more harvests per year. Calculating annual aggregate yields allows for objective comparison of cultivation systems with different harvest cycle lengths. Aggregate yield (over the whole year) might be higher with more yet shorter cycles. Which cycle length maximises yield is yet unclear and will vary from place to place depending on environmental conditions. A second important operational farm management decision is how to split up harvests into the fraction sold and the fraction replanted. A higher planting weight gives stronger growth (Pizarro and Santelices 1993) but – in cultivation systems with replanting - it implies the farmer would be selling a smaller fraction of the harvest. This may seem counterintuitive to farmers. Consider the following example. Imagine a harvest of 2 kg fresh per meter line at a selling fraction of 90%, in this scenario the farmer sells 1.8 kg m^-1^ and replants 0.2 kg m^-1^. The second farmer starts with a 4x higher replanting weight of 0.8 kg m^-1^ and harvest 3.2 kg m^-1^. This second farmer is thus selling (3.2-0.8)/3.2 = 75% of his harvest. The second farmer sells a smaller fraction of his harvest (75% vs 90%) but he sells more (2.4 kg m^-1^ vs 1.8 kg m^-1^) thanks to stronger growth at a higher replanting weight.

Throughout the growing period farmers have “fixed” costs of depreciation of material (Valderrama et al. 2013, 2015; Zuniga-Jara and Marin-Riffo 2016; Domínguez-May et al. 2017). A peak in labour costs occurs on the day of harvesting and sorting of material that will be replanted. The harvested seaweed is sorted into the two piles (sales and replants). The sales pile is placed on platforms on the beach for drying, the replant pile is sorted into individual plants^1^, which are attached to a new clean line. Finally, the same day or the next morning, the new lines are set up in sea again. If harvest costs are relatively high compared to fixed costs, then it may be more profitable for farmers to have longer cycles with less harvests per year. We should therefore consider not only yields, but also the economics.

Analysing these farm management decisions can be done experimentally, through modelling or a combination of the two. Simulation allows for easily comparing yields for many different combinations of farm management decisions. Modelling can be applied to identify optimum farm management decisions. Such optimisations will only be useful if the models are sufficiently accurate, which requires experimentation to estimate parameters and verify if predicted yields are accurately simulated. In the temperate climates advances have been made in seaweed modelling (Jackson 1987; Duarte and Ferreira 1997; Broch and Slagstad 2012; Zhang et al. 2016; van der Molen et al. 2018; Lavaud et al. 2020; Venolia et al. 2020). These models require many parameters as input, while experimental data for parameter estimation are generally lacking for the tropical seaweeds considered here. These models for temperate climates can be more complex than needed for tropical climates. In temperate climates it is necessary to model intra-annual fluctuation of temperature and irradiance, low in winter and high in summer. In tropical climates temperature and irradiance are fairly constant, so there is less need to model this intra-annual fluctuation. In the temperate climates species like *Saccharina* and *Laminaria* can grow to great depths. There it is necessary to model vertical temperature and light extinction. In tropical cultivations of the seaweeds considered here, all cultivation is near surface and the seaweeds like *Kappaphycus* and *Gracilaria* remain much smaller. There is therefore also less need to model this vertical temperature and light extinction. On the other hand, horizontal (spatial) variability is important. Much is still unknown about site suitability for tropical seaweeds. Farmers often just find out through trial and error if a site is suitable for seaweed cultivation. A more systematic approach to mapping site suitability based on environmental variables is presented by (Teniwut et al. 2019), but the scientific base for site suitability criteria is still thin.

The most commonly used model in tropical seaweed studies is the exponential growth model, Eq. 1 (Dawes et al. 1993, 1994; Hurtado et al. 2001; Kasim et al. 2016; Setyawidati et al. 2017; Periyasamy et al. 2019):

1 $${w}_f(t)={w}_{f,0}\ast {e}^{RGR\ast t}$$

In which fresh weight *w*_*f*_*(t)* increases exponentially over time *t* (expressed in days since planting) with relative growth rate *RGR* (day^-1^), starting with a weight of *w*_*f,0*_. An obvious limitation of this model is that it is only useful for describing the initial exponential growth phase of a seaweed. Species do not continue growing exponentially forever. This is reflected in lower *RGR* values reported when a seaweed is grown for a longer time (Kasim et al. 2016; Periyasamy et al. 2019). Positioned somewhere in between the complex temperate species models and the overly simple exponential model is the sigmoid model (Zuniga-Jara and Marin-Riffo 2016; Domínguez-May et al. 2017). Domínguez-May and Zuniga-Jara coupled a sigmoid biological seaweed growth model to an economic model that distinguishes between high peak labour costs at harvesting and regular costs for maintenance and depreciation. They used their model to predict the optimum harvest cycle length. Our model follows the same philosophy, with a slightly different mathematical formulation of the sigmoid. Key differences are (1) in this paper we are simulating cultivation systems with multiple harvests per year and replanting part of the harvested material, whereas Domínguez-May simulated only one cycle. And (2) here we consider simultaneously the importance of initial planting weight and harvest cycle length, whereas Domínguez-May and Zuniga-Jara did not consider effects of replanting weight. Here in this paper we also model the conversion from fresh weight to semi dry weight, and content of the harvested chemical (e.g. agar) which increases over time since planting.

There is a need for a seaweed growth model positioned somewhere between the complex models developed for the temperate climates and the too simple exponential growth model. There is a need for a model that combines the biology and economics of tropical seaweed farming. For the economic part, it is important to consider the peak in labour costs that occurs at harvesting. And there is a need for more research on how tropical seaweed growth is determined by environmental variables. We address these issues in this paper. In section 2 we present the BESeM model. In section 3 we present an experiment in which BESeM model parameters were estimated for the species *Gracilaria*. In section 4 we present simulations on management decisions on replanting weight and harvest cycle lengths.

## Bio-Economic Seaweed Model (BESeM)

The model consists of a biological part (2.1) and an economic part (2.2). Table 1 lists the model variables. Parameter estimates for the species *Gracilaria* are presented in Table 3 in the section 3 on experimental results and are discussed in the text. Table 1 shows that we express most variables in units per m^2^: kg m^-2^ and IDR m^-2^. Such an approach offers more flexibility than the common approach of expressing biomass and production costs per meter line or per plant. For example, it allows us to compare cultivation systems with the seaweed floating freely within cages (without lines) with cultivation systems with the seaweed growing attached to lines. It allows us to compare cultivation systems at different line spacings. For example, consider one site with lines of 25 m and lines running in parallel at 1 m, one line will occupy an area of 25*1 = 25 m^2^. A second site may have parallel lines at 0.8m spacing, thus the area occupied by one line will be 25*0.8 = 20 m^2^. For two farmers with the same farm size in terms of number of lines and yield per meter, the second farmer will be using a smaller area and thus have a higher yield per unit area. This higher productivity is not visible when expressing yields per unit length (meter) and does become clear when expressed per unit area (m^2^). With access to the same total area in sea (m^2^) and assuming that narrower line spacing does not negatively influence growth, the second farmer could plant 1.0/0.8 =1.25 thus 25% more lines and have higher total production, therefore also higher productivity per farmer. Calculating productivity per m^2^ offers a standardised method of comparing productivity of cultivation systems without lines and cultivation systems with different line spacings.

**Table 1:** Variables of the Bio-Economic Seaweed model

Variable	Unit	Description
*t*	Days	Days after planting
*w*_*f,g*_*(t)*	kg FW m^-2^	Gross biomass fresh from sea, at time t after planting.
*w*_*f,n*_*(t)*	kg FW m^-2^	Net biomass fresh from sea, at time t after planting
*W*_*f,n*_*(hcl,w*_*f,0*_*)*	kg FW m^-2^ year^-1^	Fresh (*f*) net (*n*) annual harvested biomass as a function of replanting weight *w*_*f,0*_ and harvest cycle *hcl*
*W*_*sd,n*_*(hcl,w*_*f,0*_*)*	kg SDW m^-2^ year^-1^	Semidried (*sd*) net (*n*) annual harvested biomass.
*cf(t)*	kg chemical kg^-1^ SDW	Concentration of the harvested chemical (e.g. Agar) in the semi-dry harvested product
*CHEM(hcl,w*_*f,0*_*)*	kg m^-2^ year^-1^	Net annual harvested chemical (e.g. Agar)
*FGP*_*sd*_*(t)*	IDR kg^-1^ SDW	Farmgate price for semi-dry seaweed as a function of *t*
*I*_*g*_*(hcl,w*_*f,0*_*)*	IDR m^-2^ year^-1^	Gross annual income per m^2^.
*PC(hcl)*	IDR m^-2^ year^-1^	Annual production cost per m^2^.
*I*_*n*_*(hcl,w*_*f,0*_*)*	IDR m^-2^ year^-1^	Net annual farm income per m^2^.

*FW* Fresh Weight, *SDW* Semi-Dry Weight, *IDR* Indonesian Rupiah’s

### Seaweed Growth Model

The sigmoid growth function was first proposed by (Verhulst 1838). The rate of fresh weight *w*_*f,g*_ increase over time (d*w*_*f,g*_/d*t*) is in this model a function of the weight *w*_*f,g*_*,* and two parameters, the maximum relative growth rate *RGR*_*max*_ and the maximum attainable weight *w*_*f,max*_:

2 $$\frac{d{w}_{f,g}}{dt}={RGR}_{max}\ast {w}_{f,g}\ast \left(1-\frac{w_{f,g}}{w_{f,\mathit{\max}}}\right)$$

Initially at a low planting weight *w*_*f,g*_ ≈0, the right term of Eq. 2 is almost 1. In this early stage the absolute growth rate is d*w*_*f,g*_/d*t* ≈ *RGR*_*max*_ * *w*_*f,g*_ and the relative growth rate is rate is $\frac{1}{w_{f,g}}\frac{d{w}_{f,g}}{dt}\approx {RGR}_{max}$. The absolute and the relative growth rate both decrease to zero as *w*_*f,g*_ approaches *w*_*f,max*_. From Eq. 2 we can derive Eq. 3 showing gross biomass (kg m^-2^) at any point in time *t* (days after planting) for a given start weight *w*_*f,0*_:

3 $${w}_{f,g}(t)=\frac{w_{f,\mathit{\max}}}{1+\left(\frac{w_{f,\mathit{\max}}-{w}_{f,0}}{w_{f,0}}\right)\ast {e}^{- rgrmax\ast t}}$$

Time *t* in the model is expressed in days after planting. For a year with multiple plantings, *t* can be calculated as:

4 $$t\;\mathit=\mathit\;mod\left(doy\mathit-{doy}_0\mathit,hcl\right)$$

Where *doy* is the Julian day of the year, *doy*_0_ is any planting day of the year, *hcl* is the cycle length including the harvest day, and *mod* is the modulo. The modulo function returns the remainder after division of one number by another. For example mod(44,45) = 44, mod(45,45) = 0, mod(46,45) = 1, mod(47,45) = 2, mod(89,45) = 44, mod(90,45) = 0, mod(91,45) = 1, etc. It generates a sawtooth timeseries in which *t* increases by 1 every day until reaching the harvest date (parameter *hcl*), drops back to 0 and then increases again by 1 every day until reaching the next harvest date. For a simulation for a year we will simulate from *doy* = 1 to 360 and we set *doy*_0_ = 1.

Of the harvested biomass *w*_*f,g*_(*t*), one part *w*_*f,0*_ is ‘replanted’ (the same day, to avoid desiccation) and the remaining part *w*_*f,g*_(*t*) *- w*_*f,*0_ is dried on platforms on the beach (Eq. 10) and stocked by the farmer until it is collected by and sold to the seaweed collector (McHugh 2003; Valderrama et al. 2013). Net fresh biomass production is therefore:

5 $${w}_{f,n}(t)={w}_{f,g}(t)-{w}_{f,0}$$

A number of growing cycles can be completed in one year. For simplicity we will consider a year consisting of 360 days. To compare productivity of cultivation systems with different harvest cycle lengths (*hcl*, days), the annual number of cycles is calculated as:

6 $$\begin{array}{ccc}N_h=360/hcl&\end{array}$$

For example, cultivation system with *hcl* = 45 days can have *N*_*h*_ 8 harvests per year, a cultivation system with *hcl* = 60 days gives *N*_*h*_ = 6 harvests per year. Total net annual production can now be expressed as the annual number of harvests *N*_*h*_ times net biomass harvested per individual cycle. At the day of harvesting, the seaweed is harvested in the morning, sorted and replanted late afternoon, or early next morning. Thus in each cycle one day is ‘lost’, i.e. the seaweed is not growing whilst not in the sea. With cycles of *hcl* = 45 days there are 8 harvests per year. The seaweed is growing in the sea *N*_h_*(*hcl*-1) = 8*(45-1) =352 days and it is on land for sorting for *N*_*h*_ = 8 days. Although missing 1 out of 45 days matters little for annual production, the theoretically correct statement is that the gross yield at harvesting is not *w*_*f,g*_*(hcl)* but *w*_*f,g*_*(hcl-1)*. We define the length of the actual growing period *lgp* as:

7 $$lgp= hcl-1$$

Annual net fresh yield is calculated from the number of harvests and the net yield at harvesting:

8 $${W}_{f,n}\left( hcl,{w}_{f,0}\right)={N}_h\ast {w}_{f,n}\left( lgp,{w}_{f,0}\right)$$

The model actually models only 1 culture cycle (Eq. 3) and the other cycles are identical replications of it (Eq. 8). We use a lower case ‘*w*’ in Eqs. 2, 3 and 5 for biomass weight at time *t* and an uppercase ‘*W*’ in Eq. 8 for annual total biomass. Combining the above equations, we can express net annual fresh production *W*_*f,n*_ as a function of the 4 parameters *hcl*, *w*_*f,*0 ,_ *RGR*_*max*_ and *w*_*f,max*_:

9 $$W_{f,n}\left(hcl,w_{f,0}\right)=\left(\frac{360}{hcl}\right)\ast\left(\left(\frac{w_{f,max}}{1+\left(\frac{w_{f,max}-w_{f,0}}{w_{f,0}}\right)\ast e^{-RGR_{max}\ast\left(hcl-1\right)}}\right)-w_{f,0}\right)$$

After harvesting, the seaweed is dried on platforms on the beach. Annual net semidry weight *W*_*sd,n*_ (kg m^-2^) is calculated from moisture contents of the seaweed fresh from sea (*m*_*f*_) and semidry (*m*_*sd*_):

10 $${W}_{sd,n}\left( hcl,{w}_{f,0}\right)=\left(\frac{1-{m}_f}{1-{m}_{sd}}\right){W}_{f,n}\left( hcl,{w}_{f,0}\right)$$

For example, consider *m*_*f*_ = 0.9 (i.e. 90% moisture fresh out of sea) and *m*_*sd*_ = 0.35 (35% moisture content after drying). The conversion factor for calculating semi-dry weight from fresh weight is then: $\left(\frac{1-{m}_f}{1-{m}_{sd}}\right)$ = 0.154. For example, a harvest of 20 kg FW (Fresh weight) will contain 0.9*20 = 18 kg water and (1-0.9)*20 = 2 kg dry matter. After drying to *m*_*sd*_ = 0.35, the semidry weight is 0.154*20 = 3.07 kg SDW, of which 3.07 * 0.35 = 1.07 kg water and 3.07*(1-0.35) = 2 kg dry matter. The example shows dry weight of 2 kg is conserved and the amount of water has decreases from 18 kg water at 90% moisture content to 1.07 kg water at 35% moisture content.

Next, we calculate the chemical fraction (content) as a function of days after planting:

11 $$cf(t)={cf}_{min}+\frac{cf_{max}-{cf}_{min}}{1+{e}^{- cf k\left(t- cf{t}_{50}\right)}}$$

This sigmoid function ranges from *cf*_*min*_ at *t*=-∞ to *cf*_*max*_ at *t*=∞; parameter *cfk* determines the steepness of the slope, *cft*_*50*_ is the time (in days after planting) at with *cf(cft*_*50*_*)* is halfway *cf*_*min*_ and *cf*_*max*_. For example if for the chemical ‘Agar’ *cf*_*min*_ = 0.1, *cf*_*max*_ = 0.2 and *cft*_*50*_ is 30, then *cf*(30) will be 0.15 (15%) at 30 days after planting. Previous studies by Periyasamy et al. (2019) and Periyasamy and Rao (2017) showed data following a sigmoid pattern for carrageenan content in semi dry *Kappaphycus* as a function of days after planting. Here we will show in section 3 the sigmoid Eq. (11) can also be used to model the concentration of the chemical agar in *Gracilaria*. In both studies, concentration increases from a relatively low content at planting to a maximum value somewhere around 40 days after planting.

Ultimately the industry is more interested in the chemical extracted from the seaweed than in the semi- dry weight. Net annual production of the harvested chemical can be calculated as:

12 $$CHEM\left( hcl,{w}_{f,0}\right)= cf(hcl)\ast {W}_{n, sd}\left( hcl,{w}_{f,0}\right)$$

### Economic model

#### Economics in the BESeM model

Our interviews with seaweed farmers in South West Sulawesi, Indonesia, suggested that farmers receive a lower price per kg semi dry seaweed when harvested early. This is consistent with the industry preferring seaweed with higher concentrations of the chemical and paying higher prices for seaweed with higher concentrations. In BESeM farmgate price is calculated as:

13 $${FGP}_{sd}(t)={FGP}_c\ast cf(t)$$

Where *FGP*_*sd*_ is the farmgate price in Indonesian Rupiah (IDR) per kg semi-dry, which is then higher when *cf(t)* is higher. *FGP*_*c*_ is a constant price per kg of the chemical *c.* Gross annual income *I*_*g*_ (IDR m^-2^ year^-1^) is calculated as:

14 $${I}_g\left( hcl,{w}_{f,0}\right)={W}_{sd,n}\left( hcl,{w}_{f,0}\right)\ast {FGP}_{sd}(hcl)$$

Substituting Eqs. 10 to 13 into Eq. 14 we can also write gross income as a function of production of the chemical:

15 $${I}_g\left( hcl,{w}_{f,0}\right)= CHEM\left( hcl,{w}_{f,0}\right)\ast {FGP}_c$$

Net income is calculated as gross income minus production costs. Production costs consist of two types of costs:

- *PC*_*m*_ = “maintenance” costs, which include material depreciation (ropes, wood, boat, nets) and labour costs (checking growth while lines are in the sea, shaking the lines to get rid of epiphytes and sediment). We refer to these costs as maintenance (*m*) costs and express them in IDR per m^2^ per day, since they occur on a continuous daily basis
- *PC*_*h*_ = “harvest” costs which is the costs that occur only at the event of harvesting. This includes the full package of labour costs that occur from harvesting to replanting: harvesting, drying, tying vegetative parts to new lines for replanting and placing the new lines back into the sea. We refer to these costs as harvest (*h*) costs and express them in IDR per m^2^ per cycle, since they occur at the end of each cycle

Total production costs *PC*(*hcl*) (IDR m^-2^ year^-1^) summed over a period of 360 days for a seaweed farm can then be calculated as:

16 $$PC(hcl)=360\ast \left({PC}_m+\frac{1}{hcl}\ast {PC}_h\right)=360\ast {PC}_m+{N}_h\ast {PC}_h$$

Where *N*_*h*_ is the number of harvests (Eq. 6). We can see that a shorter harvest cycle (*hcl*) and more frequent harvesting (*N*_*h*_) increases total costs through the right terms (1/hcl) * PC_*h*_ and *N*_*h*_ * PC_*h*_.

Finally net income, expressed in IDR m^-2^ year^-1^, is:

17 $${I}_n\left( hcl,{w}_{f,0}\right)={I}_g\left( hcl,{w}_{f,0}\right)- PC(hcl)$$

Production and income at the farm level is calculated by multiplying *W*_*n,sd*_ (kg m^-2^ year^-1^) or income *I*_*g*_ or *I*_*n*_ (IDR m^-2^ year^-1^) with farm area *A* (m^2^), which is calculated as:

18 $$A={L}_N\ast {L}_l\ast {L}_w$$

Where *L*_*N*_ is the number of lines, *L*_*l*_ is the line length (commonly 25m, range 20-30 m depending on supplier) and *L*_*w*_ is the width or spacing between the lines (commonly 1.0m). Multiplying *I*_*n*_ with *A* gives the net income per farm per year. In a similar vein, we may want to convert from weight in kg m^-2^ to weight per plant. Let one line hold *L*_*pd*_ plants per meter, the plant density per m^2^ is *PD = L*_*pd*_ */ L*_*w*_ plants m^*-2*^. Plant weight in gram per plant can be calculated as 1000 * *w / PD* where the 1000 is for conversion from kg to gram. A plant weight of 30 g per plant at *L*_*w*_ = 0.75 m and *L*_*pd*_ = 5 plants m^-1^ corresponds with 30 * 5 / 0.75 = 200 g m^-2^ = 0.001 * 200 = 0.2 kg m^-2^. And 0.2 kg m^-2^ will have 5 / 0.75 = 6.67 plants m^-2^, so each individual plant will have a weight of 1000 * 0.2 / 6.67 = 30 g plant^-1^

#### Recurring operating costs and investments

In BESeM we classified production costs into two categories, “maintenance” and “harvesting”. Zuniga-Jara and Marin-Riffo (2016) presented a different set of economic parameters, with “Recurring operating costs” and “Investments”. Table 2 summarises the differences between the two approaches. “Recurring operating costs” is in the BESeM approach split into two categories of costs, those with are continuous (Shaking of lines to get rid of epiphytes, depreciation of material) and those which are event based, i.e. only occurring at the event of harvesting (with a peak in labour costs, see Table 2). This distinction between continuous and event based is important because recurring operating costs will be higher in case of a production system with more frequent harvesting. A second important difference is that in BESeM any continuous cost, whether continuous operating cost or continuous costs of depreciation of investment is added to the same category of “Maintenance cost”. In future elaborations of the BESeM model it may be useful to further split the economic costs into more categories than the ones considered here and to consider depreciation of material and re-investments over time. Such further elaborations would also enable calculating Net Present Value (NPV), which is an indicator of how much value an investment or project adds to the firm (Zuniga-Jara and Marin-Riffo 2016). Such further economic elaborations are beyond the scope of the current paper.

**Table 2:** Two approaches to economic costs

	(Zuniga-Jara and Marin-Riffo 2016)	
BESeM	Recurring operating costs	Investments
Maintenance costs	Shaking of lines to get rid of epiphytes	Depreciation of material Re-investments
Harvest costs	Labour costs of harvesting Labour costs of drying on beach Labour costs of tying plants to new lines	

## Experiment

### Material and Methods

#### Objectives & design

The objectives of the experiment were to (1) estimate model parameters from a site where Gracialaria is actually grown and (2) assess if and how model parameters depend on environmental conditions.

To achieve these objectives, an experiment was conducted in Takalar, South West Sulawesi, Indonesia. Important features of the experiment were to (1) monitor growth for prolonged time (120 days) so that the critical parameter *w*_*f,max*_ could be properly estimated and (2) conduct the experiment in 6 sites with contrasting environmental conditions, to establish how parameters *RGR*_*max*_ and *w*_*f,max*_ depend on environmental conditions. Intentionally a relatively low initial plant weight *w*_*f,0*_ was chosen, to ensure that the experiment would start in the initial exponential part of the growth curve and not immediately in the linear part of the growth curve.

**Fig. 1:**
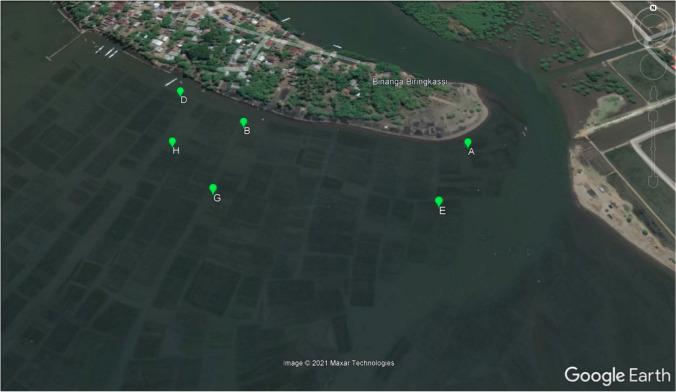
Sampling sites. Screenshot from Google Earth. Sites were positioned along the coast of a tiny peninsula in Takalar, SW Sulawesi, Indonesia (at 119^o^23’38.4E, 5o27’27.36S). Sites A and E are closest to the river mouth. Seaweed fields in are clearly visible and are all 25 m wide (standard line length), planted in parallel along the coast

Figure 1 shows the location of the sites. Sites were positioned 0, 300 and 400 meters along the coast, Northwards starting from a river mouth and at two positions out of shore, respectively 30 and 100 m perpendicularly to the shore. Location for the 6 lines was rented from the local seaweed farmer. At each site a line of standard length 25 m was planted. Plant density was 5 plants m^-1^, thus in total each line held 125 plants. Parallel lines were spaced 0.75 m apart. Seaweed was planted on 15 June 2021. Additionally, 125 plants were separately weighed to check if (as planned), plant weight was 10 g fresh per plant.

#### Measurements

**Fig. 2:**
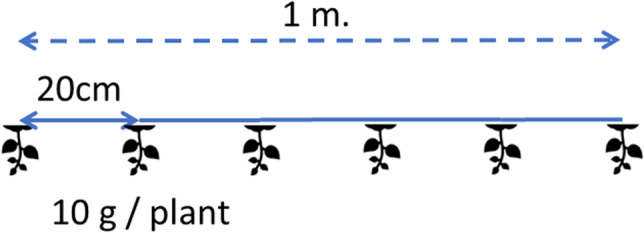
Plant distance

**Fig. 3:**
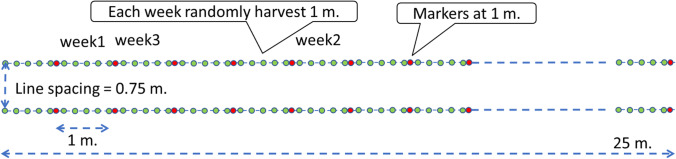
Line setup

Samples are taken once every 7 days up to day 49 after planting and thereafter once every 14 days. For *Gracilaria* each sampling date randomly 1 meter (on a line of 25m) was sampled. The 5 plants on each sampled meter were treated as replicates. See Figs. 2 and 3 for illustration of the biomass sampling scheme. Each meter (5 plants) from each site was packaged in plastic, placed in a cooling box and transported to the laboratory. At the laboratory each plant was cleaned, dried with paper towels and weighed (fresh weight) and then dried in an oven for 5 days at 60^o^C. Moisture content fresh (*m*_*f*_) was calculated as 1 - dry weight / fresh weight. Agar content was determined using standard methods but could not be determined in earlier dates due to too small sample sizes.

Each sampling date per site 3 measurements (replicates) were made of a series of environmental variables. Some measurements could be taken on site, for others 3 bottles were sampled per site and taken to the UNHAS laboratory for analysis. Table 3 lists the environmental variables and the methods used to measure them.

**Table 3:** Seawater physical and chemical parameters and methods/equipment used

No	Parameter	Materials	Methods
1	Temperature	Hanna Multiparameter Model HI 98194	In situ
2	Salinity	Hanna Multiparameter Model HI 98194	In situ
3	pH	pH meter portable Model PH-201	In situ
4	Dissolved oxygen (DO)	Hanna Multiparameter Model HI 98194	In situ
5	Phosphate (PO4)	Genesys 150. UV-Visible Spectrophotometer	Laboratory
6	Nitrate (NO3)	Genesys 150. UV-Visible Spectrophotometer	Laboratory
7	Current velocity	Marotte HS Drag-Tilt Current Meter	In situ
8	Clarity	Secchi disk	In situ

On 3 occasions when seaweed collectors visited the village, 3 semi-dry seaweed samples were taken from the material sold to the collectors, 9 in total. Semi-dry seaweed was further dried to 0% moisture in the lab and the parameter moisture content semi-dry (*m*_*sd*_) was calculated as 1 – dry weight / semi-dry weight.

#### Parameter estimation

Parameters *RGR*_*max*_ and *w*_*f,max*_ (Eq. 3) were estimated using function *nls* in the statistical package ‘r’ (R_Core_Team 2021). *w*_*f,max*_ was converted from maximum fresh weight in gram per plant to maximum fresh weight in kg per m^2^ by multiplying with 0.001 * 5 / 0.75, where 0.001 is for conversion from gram to kg, 5 is the number of plants and 0.75 is the distance between lines. Parameters *RGR*_*max*_ and *w*_*f,max*_ were estimated 7 times: once for a model fitted all data together, and once for each site separately. Parameters for the agar content as a function of days after planting (Eq. 11) were estimated with the *optim* function in the statistical package ‘r’ (R_Core_Team 2021).

Per site, mean and standard deviation of environmental variables was calculated. Results indicated no temporal trend in the environmental variables. To analyse if and how parameters *RGR*_*max*_ and *w*_*f,max*_ of the 6 sites were dependent on environmental variables we plotted these two parameters on the y-axis with on the x-axis the environmental variables, their mean and the error bars. Significance of the relation (if any) was visually assessed.

### Results

#### Biomass & environment

**Fig. 4:**
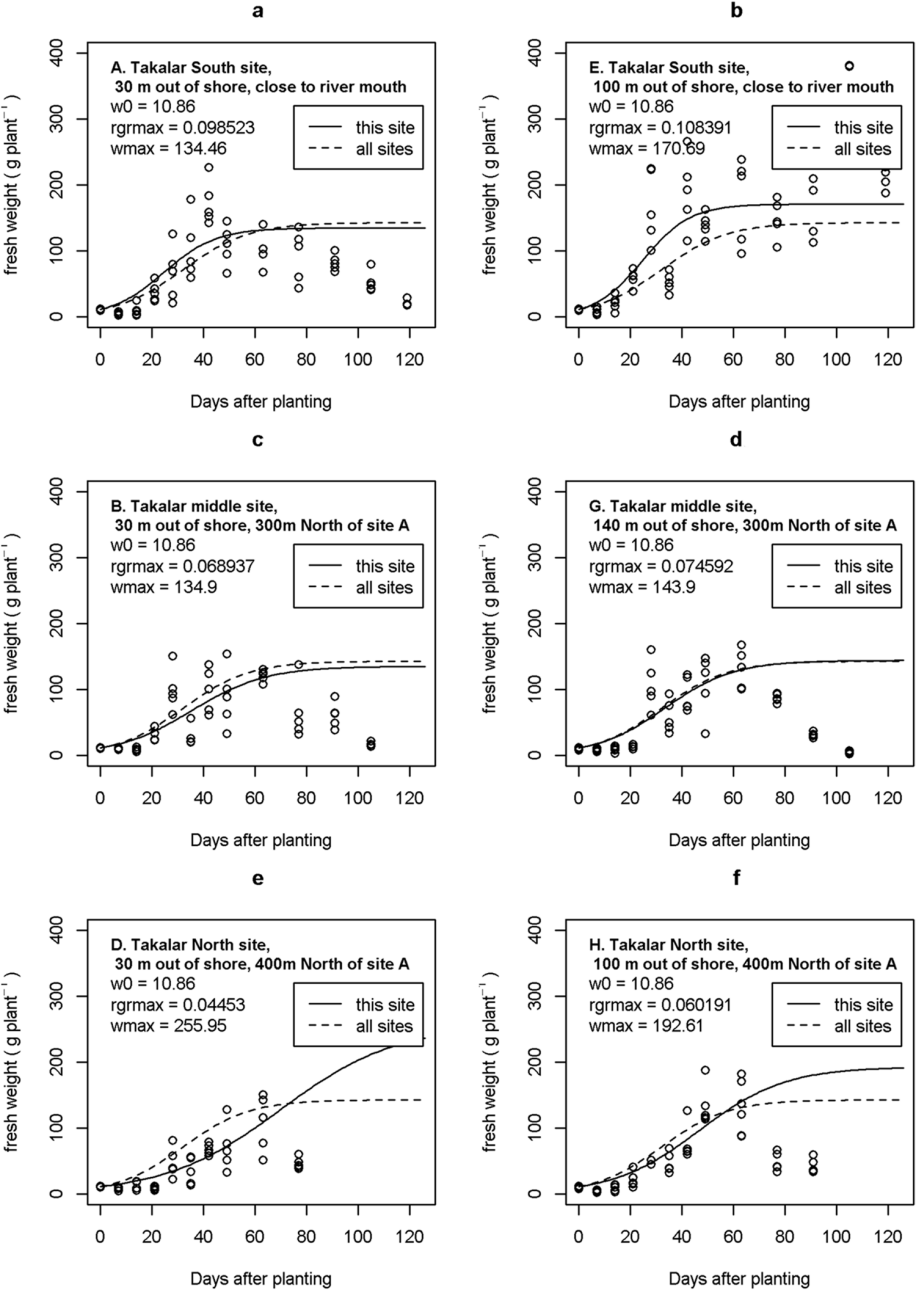
Observed and simulated plant weights at the 6 sites

**Fig. 5:**
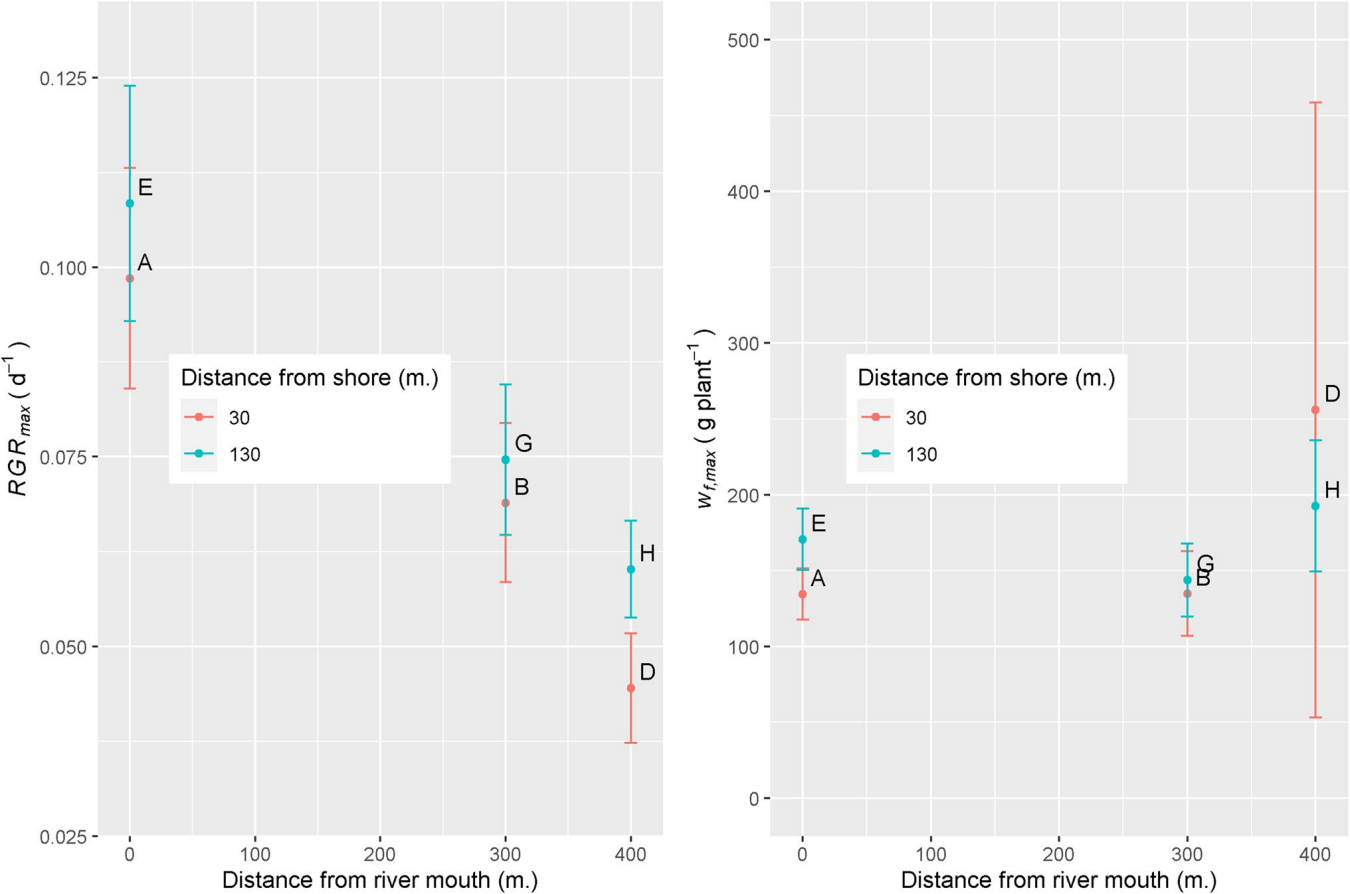
Relation between sigmoid growth parameters and site distance from river and distance from shore. Error bars show the standard error of the estimated parameter. Upper case letters A-H refer to sites shown in Fig. 1

Average plant weight at the start of the experiment, calculated from 125 samples, was *w*_*f,0*_ = 10.86 g fresh per plant, slightly higher than the planned 10 g per plant. Figure 4 shows observed and simulated plant weights over time. In site E (Fig. 4b) we did find a sigmoid for the entire 120 days. In the other 5 sites a sigmoid reached its’ plateau at 63 days after planting. Observations in these 5 sites showed an unexpected biomass decline in biomass after 63 days. The cause of this decline was not understood. Up to 63 days growth was sigmoid and sigmoid models were fitted on observations from 7 to 63 days. Large scatter of observations around the calibrated model lines is seen in all sites. The dashed line shows the model for all sites together, the solid line shows the model separately fitted per site. In Fig. 4a, b, the solid line is above the dashed line, indicating growth in the two sites near the river mouth (sites A&E) is stronger than average (dashed line). In Fig. 4e, f, the solid line is below the dashed line, indicating in the two sites furthest away from the river mouth (sites D&H), growth is less strong than average (dashed line). Figure 5a indicates growth closer to the river mouth is stronger due to higher *RGR*_*max*_: *RGR*_*max*_ decreases from around 0.10 near river mouth to around 0.05 at 400m from the river mouth. These *RGR*_*max*_ parameter values are in the same range as normally reported in studies on the initial exponential growth phase of tropical seaweeds (Dawes et al. 1993, 1994; Hurtado et al. 2001; Kasim et al. 2016; Setyawidati et al. 2017; Periyasamy et al. 2019). Figure 5b shows stronger growth closer to the river mouth is not due to higher *w*_*f,max*_, for which Fig. 5b shows insignificant differences between sites. Figure 5 further shows no significant effect of distance from shore and it shows that for the two sites furthest away the river mouth (sites D&H), estimates of parameters *w*_*f,max*_ are most uncertain. For environmental variables we first analysed if there was a temporal trend – there was none (result not shown). This is we believe a normal situation in much of the tropical near-shore marine environments. Boxplots of environmental variables per site indicated no significant site differences for any of the sites (result not shown). Consequentially, sigmoid growth parameters *RGR*_*max*_ and *w*_*f,max*_ were not correlated with environmental variables (Table 4).

**Table 4:** Parameter estimates for seaweed species *Gracilaria*

Parameters	Unit^1^	Value	Description	Source
*RGR*_*max*_	g g^-1^ day^-1^	0.0778	Relative growth rate	Experiment, Fig. 4, dashed line (all sites)
*w*_*f,max*_	kg FW m^-2^	0.951113	Maximum fresh weight	Experiment, Fig. 4, dashed line (all sites). w_*f,max*_ was 142.667 g plant^-1^. Converted to kg m^-2^: 0.951113 = 0.001 * 142.667 * (5 / 0.75) ; conversion kg / g * g / plant * (plants / m line / distance between lines (m)).
*cf*_*min*_	kg agar kg^-1^ SDW	0.0831	Minimum chemical content at *t=-∞*	Experiment, Fig. 6. The chemical here in this study is ‘Agar’
*cf*_*max*_	kg agar kg^-1^ SDW	0.202	Maximum chemical content at *t=+∞*	Experiment, Fig. 6
*cfk*	day^-2^	0.180	Steepness of slope with which content increases with days after planting	Experiment, Fig. 6
*cft*_*50*_	Day	29.5	Days after planting at which chemical content is halfway between *cf*_*min*_ and *cf*_*max*_	Experiment, Fig. 6
*m*_*f*_	g g^-1^	0.872217	Moisture content fresh from sea	Experiment
*m*_*sd*_	g g^-1^	0.293	Moisture content semidried (dried on platform on the beach by farmer)	Experiment
*w*_*f,0*_	kg FW m^-2^	0.0724	Fresh biomass weight replanted.	Experiment, Fig. 4, dashed line (all sites). w_*f,0*_ was 10.86 g plant^-1^. Converted to kg m^-2^: 0.0724 = 0.001 * 10.86 * (5 / 0.75). In the simulations we also considered different values for this parameter.
*hcl*	days	45	Harvest cycle length: days from planting to harvesting, including the harvest day	45 is common farmers’ practice. In the simulations we also considered different values for this parameter.
*FGP*_*c*_	IDR kg^-1^ agar	30,769	Farmgate price of the chemical, in this case agar.	See calculations in Table 5.
*PC*_*m*_	IDR m^-2^ day^-1^	0.658	Production cost for maintenance (m). This is includes depreciation of material plus daily maintenance of lines in sea	See calculations in Table 5.
*PC*_*h*_	IDR m^-2^ cycle^-1^	386.7	Production cost at harvesting (h). This is the sum of costs of harvesting + drying + replanting	See calculations in Table 5.
*L*_*N*_		600	Number of lines per farm	Authors’ interviews. Range 200-1000
*L*_*l*_	m	25	Length of a line	Authors’ observations. Standard line length. See Figs. 2 and 3 for illustration.
*L*_*w*_	m	0.75	Width = spacing = distance between lines	Authors’ observations. See Figs. 2 and 3 for illustration.
*L*_*pd*_	Plants m^-1^	5	Line plant density, plants per meter	Experiment, and farmer’s practice

^1^In the units, FW indicates Fresh Weight, SDW is Semidry Weight (dried on beach), IDR is Indonesian Rupiah

#### Agar & moisture content

**Fig. 6:**
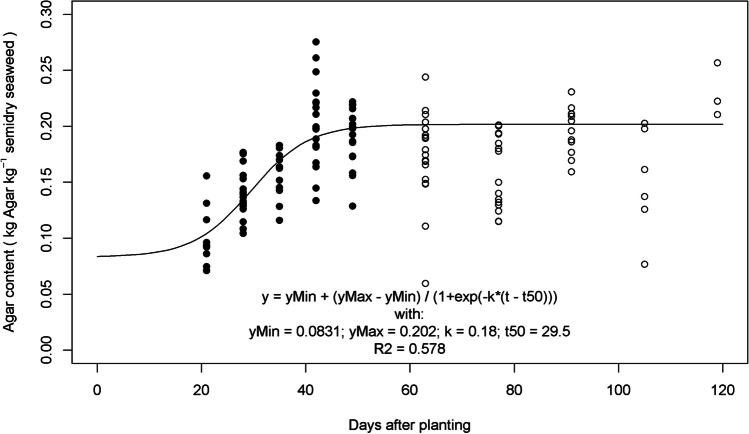
Agar content (kg agar kg^-1^ SDW) increases with days after planting. The model was fitted on the solid dots. Agar was not measured before 21 dap because samples were too small for extraction.

Figure 6 shows Agar content increased with days after planting. The sigmoid model showed a better fit when fitted on the data up to 49 days after planting (dap), so the sigmoid model was fitted on those data. Although the model describes much of the variation in the observations (R^2^=0.578), we can also see much variation along the predicted line. Agar curves per site (not shown) did not differ significantly from the general relation shown in Fig. 6.

Mean moisture content of the fresh and semi-dry seaweed was *m*_*f*_ = 0.872 (estimated from all data shown in Fig. 4) and *m*_*sd*_ = 0.293 (estimated from semi dry samples sold to seaweed collector).

#### Economic parameters

Table 5 shows how we derived BESeM economic parameters from a detailed economic study for the study site by (Tahang et al. 2019). They report a detailed economic analysis at the farm level. In Table 5 we translate detailed economic production costs from farm level to costs per square meter (m^2^). Interestingly, Tahang et al. (2019) do not report on farm size; we estimated farm size through observations made by dr Latama, co-author of both the BESeM paper and the study by Tahang et al. (2019).

**Table 5:** Derivation of production BESeM economic parameters for seaweed species *Gracilaria* in Takalar region, 2019

	Quantity	Unit	Source
A. Farm size			
Lines per farm (parameter *L*_*N*_)	600		Gunarto Latama, pers.comm.
Length of a line (parameter *L*_*l*_)	25	m	Gunarto Latama, pers.comm.
Distance between lines (param. *L*_*w*_)	0.75	m	Gunarto Latama, pers.comm.
Farm size (*A*)	11,250	m^2^	Authors' calculation (Eq. 18): 600 * 25 * 0.75
B. Maintenance costs			
Depreciation of material (lines, boat,etc)	2,350,000	IDR farm^-1^ year^-1^	"Depreciation" as reported in table 2 in Tahang et al. (2019). In their text Tahang explain depreciation is calculated as sum of depreciation costs of boat, lines, bottles, etc
Maintenance labour costs	350,000	IDR farm^-1^ year^-1^	"Maintenance" as reported in table 2 in Tahang et al. (2019)
Total	2,700,000	IDR farm^-1^ year^-1^	Authors' calculation: 2,350,000 + 350,000
Maintenance costs (parameter *PC*_*m*_)	0.658	IDR m^-2^ day^-1^	Authors' calculation: 2,700,000 / 11,250 / 365
C. Harvesting costs			
Harvesting costs (incl harvesting & separation into replant & sales pile & drying)	11,200,000	IDR farm^-1^ year^-1^	Sum of "harvest" + "harvesters" + "dryer" as reported in table 2 in Tahang
Binding seedlings to lines	10,550,000	IDR farm^-1^ year^-1^	Sum of "seeds" + "seedling binder" as reported in table 2 in Tahang
Total harvesting costs	21,750,000	IDR farm^-1^ year^-1^	Authors' calculation: 11,200,000 + 10,550,000
Number of harvests / year	5		Tahang et al. (2019)
Harvesting costs (parameter *PC*_*h*_)	386.7	IDR m^-2^ harvest^-1^	Authors' calculation: 21,750,000 / 11,250 / 5
D. Farmgate price			
Farmgate price for semidry seaweed, harvested at 45 days (parameter *FGP*_*sd*_(45))	6,000	IDR kg^-1^ semidry seaweed	seaweed farmers’ cooperative Kospermindo in Makassar Indonesia, price in year 2019
Agar content when harvested at 45 days	0.195	Kg agar kg^-1^ semidry seaweed	Authors’ experiments, Fig. 6. In Eq. (11) filling in *t*=45 we obtain *cf*(45) = 0.195
Farmgate price for agar (parameter *FGP*_*c*_)	30,769	IDR kg^-1^ agar	Authors' calculation. From Eq. 13: *FGP*_*sd*_(t) = FGPc * cf(t). For harvest cycle length 45 days, filling in *t* = 45 and re-writing we obtain: FGPc = *FGP*_*sd*_(45) / *cf*(45) = 6,000 / 0.195 = 30,769

Table 5 shows how the “maintenance” costs *PC*_*m*_ are calculated from labour costs and costs of depreciation of materials. What is called “harvesting” costs (*PC*_*h*_) in the BESeM model is in fact the sum of all (mainly labour) costs that occur at the event of harvesting. *PC*_*h*_ includes both the costs of harvesting and the costs of replanting. A more detailed economic breakdown of these costs is found in Tahang et al. (2019). For example, they report investment costs and depreciation costs of boat, main rope, small rope, buoys (plastic bottles) and sheeting. The BESeM parameters *PC*_*m*_ and *PC*_*h*_ are thus aggregated values that are calculated from more elaborate economic analyses.

Since seaweed farming is often a small scale family labour business it can be difficult to estimate labour costs: husband does not pay wife for tying plants to lines and wife does not pay husband for harvesting. A common scientific solution in this context, also followed by Tahang et al. (2019), is to take opportunity costs or wage labour costs as an estimate for family labour costs (Valderrama et al. 2013, 2015). Opportunity costs were affected by the COVID-19 pandemic. The pandemic lead to job losses in the tourism sector, which in turn lead to greater supply of wage workers and in turn lower wages. Here for consistency with the normal situation we used pre-COVID labour costs as reported in Tahang et al. (2019)

Starting with a known farmgate price expressed in IDR per kilogram semidry seaweed, and our estimate (Fig. 6) of agar content at the normal 45 days harvest cycle length, we calculate the farmgate price expressed in IDR per kilogram agar, i.e. the BESeM parameters *FGP*_*c*_. Farmgate prices are subject to economics of price and demand. Part of the people working in the tourism sector losing their jobs have moved into seaweed farming. This has led to increased supply, which has in turn had a negative effect on farmgate prices (Langford et al. 2021). According to seaweed farmers’ cooperative Kospermindo in Makassar Indonesia, farmgate prices for *Gracilaria* have dropped from 5,000-7,000 IDR kg^-1^ semidry in 2019 to 3,800-4,000 IDR kg^-1^ semi-dry in 2021. For consistency with the production cost parameters, which were also derived pre-COVID (Tahang et al. 2019), we used in our simulations a farmgate price of from *FGP*_*sd*_(45) = 6,000 IDR kg^-1^ semidry.

## Simulation

### Harvest cycles

**Fig. 7:**
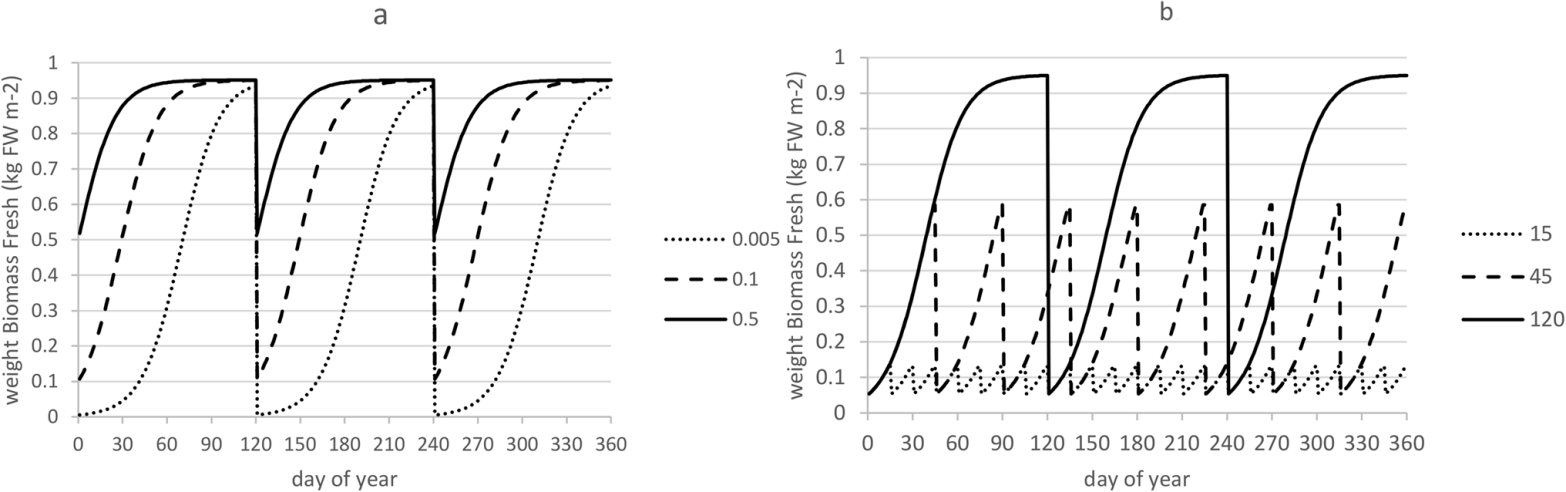
Growth cycles, with **a** three different start weights, (*w*_*f,0*_ = 0.005, 0.1 and 0.5 kg FW m^-2^) and **b** three different harvest cycle lengths (*hcl* = 15, 45 and 120 days)

Figure 7a illustrates the sigmoid growth curve at three different start weights for cycles of 120 days, simulated with parameters *RGR*_*max*_ and *w*_*f,max*_ derived from the experiment and listed in Table 4. With a low start weight a sigmoid curve is shown in which growth is first exponential, then linear and then flattening off towards *w*_*f,max*_. With higher start weights growth is immediately linear and the plateau of *w*_*f,max*_ is reached earlier. Figure 7b illustrates multiple harvest cycles for a fixed replanting weight of 0.05 kg FW m^-2^ at different harvest cycle lengths. Longer cycles give higher production per cycle, but less harvests per year. Table 6 illustrates how annual net yield can be calculated from Fig. 7b. In this example for the replanting weight of *w*_*f,0*_ = 0.05 kg FW m^-2^, Table 6 shows highest yield per cycle for the 120 days cycle and highest net annual yield for the 45 days cycle. Different results are obtained when also *w*_*f,0*_ is varied. In the next section we will jointly optimise the harvest cycle length *hcl* and the replanting weight *w*_*f,0*_.

**Table 6:** Calculation of aggregate annual net yields from Fig. 7b

Cycle (days)	Harvests / year	Yield (kg FW m^-2^ per cycle)		Yield (kg FW m^-2^ per year)		Fraction sold (%)
		Gross	Net	Gross	Net	
15	24	0.13	0.08	3.14	1.94	62%
45	8	0.59	0.54	4.68	4.28	91%
120	3	0.95	0.90	2.85	2.70	95%

Note at *hcl* = 120, the yield per cycle of 0.95 corresponds with the parameter *w*_*f,max*_ = 0.951 in Table 3

### Optimisation

**Fig. 8:**
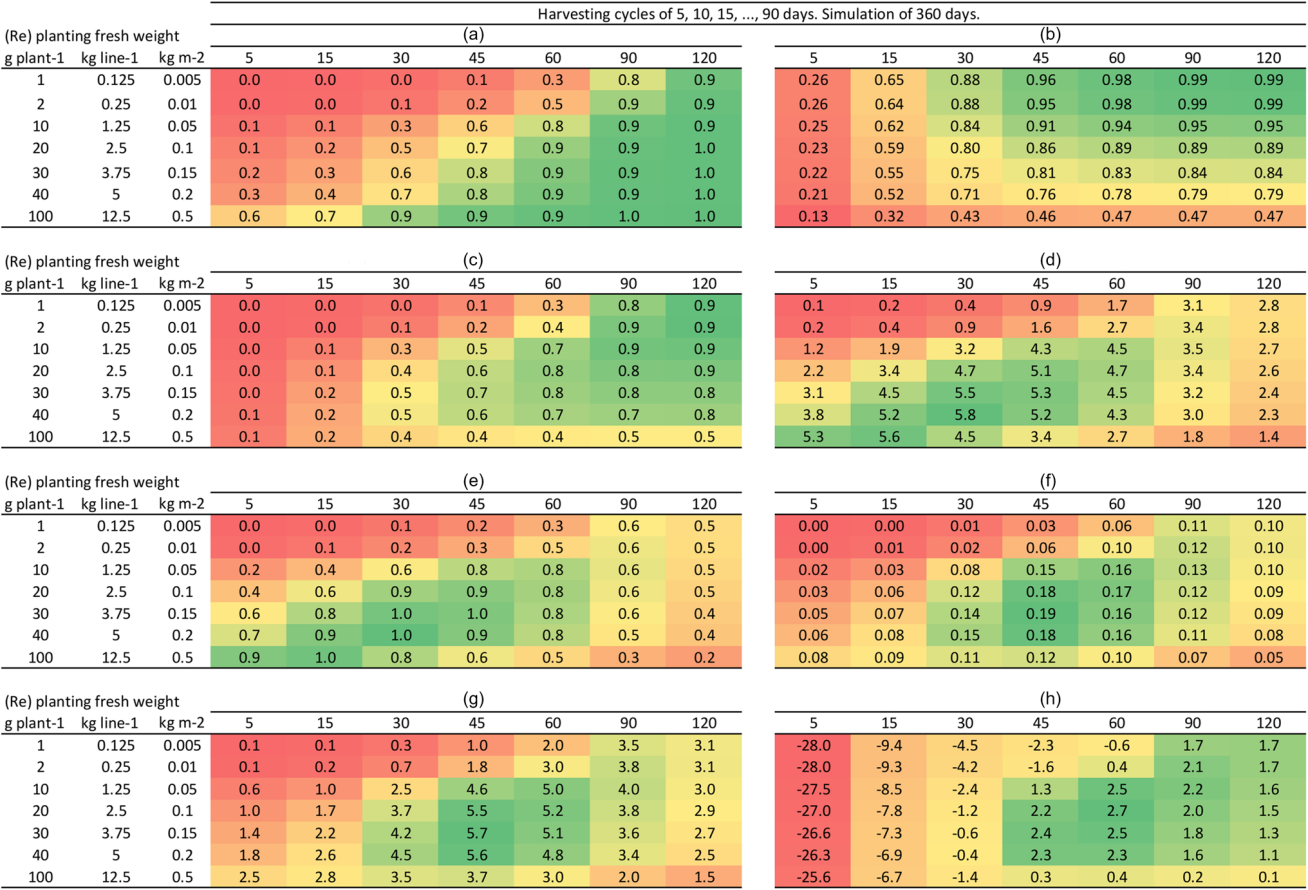
Scenarios of production and income as a function of replanting weight (*w*_*f,0*_, in the rows) and harvest cycle length (*hcl*, in the columns)

The BESeM model was run 7*7=49 times, at 7 different harvest cycle lengths (*hcl* = [5, 15, 30, 45, 60, 90, 120] days) and 7 different replanting weights (*w*_*f,0*_ = [0.005, 0.01, 0.05, 0.10, 0.15, 0.2, 0.5] kg FW m^-2^; Corresponding replanting weights in gram FW plant^-1^ are respectively [1, 2, 10, 20, 30, 40, 100]). For any given combination of *w*_*f,0*_ and *hcl*, we present simulated yields and income in Fig. 8. We discuss the results:

1. Figure 8a shows gross yields per cycle. In the line with replanting weight of 0.05 kg FW m^-2^ and in the columns for *hcl* 15, 45 and 120, Fig. 8a shows the same gross yield per cycle as reported in Table 5: 0.1, 0.6 and 0.9 kg FW m^-2^. Not surprisingly, highest yields per cycle are obtained in the bottom right corner, i.e. with high replanting weight and long cycles.
2. Figure 8b shows which fraction of the harvest is sold. In the line with replanting weight of 0.05 kg FW m^-2^ and in the columns for *hcl* 15, 45 and 120, Fig. 8b shows the same fractions sold as in Table 5: 0.62, 0.91 and 0.95.
3. Figures 8c and d show net yields per cycle and per year. For a replanting weight of 0.05 kg m^-2^ (=10 gram fresh per plant) and at a 45 days harvest cycle, net annual yield is 4.3 kg FW m^-2^ year^-1^ (see also Table 5). Annual aggregate net yield is highest for a medium replanting weight of 30-40 g per plant and short cycles of 30 days.
4. Figures 8e and f show net annual production converted to semi-dry (Fig. 8e) and agar (Fig. 8f). From 8e to 8f, we see the optimum cycle length shifting to the right (from 30 day optimum in 8e to 45 days optimum in 8f). This shift occurs due to lower Agar content at early harvesting (Fig. 6).
5. Figure 8g shows gross income in IDR m^-2^ year^-1^ is maximised with a harvest cycle of 45 days and a replanting weight of 30 g plant^-1^. In this scenario, 81% of the gross harvest is sold (Fig. 8b) and 19% is replanted.
6. Finally Fig. 8h shows simulated net income in IDR m^-2^ year^-1^. Comparing Fig. 8g (gross income) with Fig. 8h (net income), one can see the optimum replanting weight *w*_*f,0*_ shifts from 30 to 20 g plant^-1^ and the optimum harvest cycle length *hcl* shifts from 45 to 60 days. The shift towards less and longer cycles is caused by the high costs of harvesting operations relative to maintenance costs. The higher the harvesting costs *PC*_*h*_ relative to maintenance costs *PC*_*m*_, the more the optimum shifts to the top right in Fig. 8h.

An important message from our interpretation of Fig. 8 is that it is really the combination of start weight and cycle length that matters. Step by step, our discussion of these figures reflects the rationale with which the BESeM model was developed, factoring in decisions on replanting and harvesting, aggregation to the annual level, accounting for Agar concentration which increases over time towards a plateau and accounting for the high labour costs that incur at harvesting/replanting time.

## Discussion

### Main findings

We presented a new model for tropical seaweed farming. The sigmoid model (Eqs. 2 and 3) is a logical and necessary extension of the much used exponential model (Eq. 1). The BESeM model considers the biology and economics, the common practice of replanting part of the harvest and the high labour costs around harvesting time.

Biological model parameters were estimated from an experiment, economic parameters were estimated from literature and interviews. It served to test the model and to illustrate how parameters can be estimated. Important features of the experiment were that biomass was monitored for 120 days, which is necessary for estimating key model parameter *w*_*f,max*_. To our best knowledge this is the first study to monitor tropical seaweed growth for such a long time. Earlier studies monitored growth for 45 days or less, which is sufficient for studying initial expo-linear growth but not enough for researching when and to what maximum weight growth flattens off. Especially if start weights are low, it takes more than 45 days to reach the plateau of *w*_*f,max*_. Data did not show a clear relation between biological growth parameters and environmental variables. This comes as a surprise and it may be of interest to conduct similar experiments in more sites. We discuss this finding in more detail below.

A series of simulations was presented to illustrate how model outcomes respond to two key farmers’ management parameters, the replanting weight and the harvest cycle length. Simulation outcomes suggests there may be an optimum combination of replanting weight and harvest cycle length at which gross and net income is maximised. The comparison of the optima for gross and net income highlights the high economic costs of harvesting/replanting operations. In this study, Fig. 8 suggests gross income is maximised with harvest cycle lengths of 45 days, while net income is income is maximised with harvest cycle lengths of 60 days (thus with less harvests per year). The analysis of production costs certainly requires further scrutiny, but in any case, the work presented here shows how these costs can be factored in.

It seems too early at this stage to use the model simulation outcomes (Fig. 8) for farm management recommendations. We still understand too little about how environment affects growth and we are still quite uncertain about model parameters, especially the production costs. We presented a detailed analysis of production costs and of farmgate price. We acknowledge that both production costs and farmgate prices are both uncertain and vary between years and sites. For future studies it may be of interest to elaborate the economic part of the BESeM model. We discuss these uncertainties and possible extensions in more detail in section 5.2.2 of the discussion. We found during field visits farmers often try cultivating species like *Kappaphycus* for which farmgate prices are much higher than for *Gracilaria*, around 23,000 and 6,000 IDR kg^-1^ SDW in 2019 for the two species respectively. Farmers reported that sometimes, for unknown reasons, simply the *Kappaphycus* will not grow. Finding out which seaweed will grow where is often a matter of trial and error. In some cases, farmers cultivate only 1 species, in other sites they cultivate multiple species. We also reported on great uncertainty in production costs. The net income estimates in Fig. 8 therefore should not be taken as an accurate estimate of farmers’ income – there is simply yet too much uncertainty to make such estimates. The fact that parameters are hard to estimate does not invalidate the approach as such.

### Uncertainties, need for further research

#### Biological parameters

Biological parameters were estimated from an experiment and showed large uncertainty. Moreover, the results showed three unexpected results. Firstly, our hypothesis on sigmoid growth was contradicted by the results of the experiment. Instead of reaching and staying at a maximum weight, the data showed for 5 out of 6 sites a sigmoid up to 63 days (expected) followed by a decline of biomass after 63 days (unexpected). The cause of this decline remains unclear. Further research is needed to replicate this finding and, if it persists, to identify the cause of decline after peaking. Secondly, the experimental data (Fig. 4) showed at 7-14 days after planting a small reduction in biomass compared with the start weight. Exponential growth seemed to start with some delay. Possibly including a ‘transplanting shock’ or ‘lag effect’ for this initial phase could further improve the model. This first should be tested in experiments. Thirdly we obtained mixed results from our analysis of effects of environment on model parameters. We did find one model parameter, *RGR*_*max*_, correlated with distance from river mouth. Based on common knowledge and on a literature review (Table 7) we expected to find a decline in nutrient concentrations moving away from river mouth. We did not find such gradients in our dataset. Consequentially, we also did not find a relation between parameter *RGR*_*max*_ and marine nutrient concentration. One new hypothesis that arose during the research is that possibly river outflow diverts up North alongside the coast (Fig. 1) and flows much less perpendicular out of the coast into the open sea. If true that would explain why we did not find significant differences in nutrient concentrations between our 6 sites. At the same time it leaves the question unanswered on why *RGR*_*max*_ was higher near the river mouth.

**Table 7:** Indonesia studies on coastal nutrient concentrations

Inshore (0-0.5 km) at estuary or mangrove^a^			Near/off-shore (0.5-5.0 km from shore)^a^		
Study	Nitrate (μmol L^-1^)	Phosphate (μmol L^-1^)	Study	Nitrate (μmol L^-1^)	Phosphate (μmol L^-1^)
Damar (2012)	2.13		Kegler et al. (2018)	0.15	0.12
Amien et al. (2020)	3.14	1.07	Baohong et al. (2016)	0.55	0.03
Pratiwi et al. (2018)	3.14	0.21	Jennerjahn et al. (2004)	0.68	0.28
Maslukah et al. (2019)	5.07	4.82	Kasim et al. (2016)	0.78	0.06
Dong et al. (2011)	9.15		Rahadiati et al. (2017)	0.93	0.79
Jennerjahn et al. (2004)	32.20	1.20	Yulianto et al. (2017)	0.97	0.84
Average	9.14	1.82	Average	0.68	0.35
Median	4.10	1.13	Median	0.73	0.20

^a^The calculated averages from these studies and the classification into “inshore” or “near/off-shore” is ours. All reported values were converted to μmol L^-1^ using molar mass of NO_3_ and PO_4_

As an extension to the BESEM model one could replace the fixed *RGR*_*max*_ in Eqs. 2 and 3 with *RGR*_*max*_ *(x) = b*_*0*_ *+ b*_1_*x, where *x* is the distance from river mouth and *b*_*0*_ and *b*_*1*_ could be estimated from the data points in Fig. 5. In theory such a relation could be used for extrapolation (still further away from our river mouth, or for making predictions near other river mouths). However, we since the cause of the *RGR*_*max*_ ~ river mouth relation remains unclear and since every river is different, we would caution not do such extrapolations. Instead, we recommend more research. Before starting the biomass sampling, one could first do an environmental sampling to pre-select contrasting sites and only thereafter simultaneously monitor seaweed and environment in these more contrasting sites.

#### Economic parameters

We showed there is much uncertainty about the economic parameters. Two studies provide recent site-specific economic data for our site (Limi et al. 2018; Tahang et al. 2019). One particular issue is that in BESeM economic production cost parameters are expressed in IDR square meter (IDR m^-2^) while these two studies report production at the farm level without reporting farm size. These studies do not present net income such as calculated in Fig. 8 in this paper (per m^2^ per year). Consequentially, it was impossible to compare simulation outcomes (Fig. 8) with values reported in these two economic studies.

The more extensive papers on seaweed farming economics in 6 tropical countries by (Valderrama et al. 2013, 2015) do report farm size in terms of total length of lines, number of cycles per year and a breakdown of costs. As we showed in Table 5, it is possible to classify these various costs (investment, re-investiment, opportunity costs) into the two aggregate costs *PC*_*h*_ and *PC*_*m*_ as used in the BESeM model. Detailed economic studies are thus essential for estimating our aggregate costs parameters *PC*_*h*_ and *PC*_*m*_. Valderrama et al. (2013, 2015) present one figure suggesting a distance between lines of 1 m, from personal observation we found it ranges between 0.5 to 1.0 m; on average on our site spacing between lines was 0.75 m (Table 4, parameter *L*_*w*_). Clearly this parameter has large impact on calculated farm area (Eq. 18) and so also yield measurements and production costs per unit area depend on this parameter (Table 5). For accurate calculation of parameters *PC*_*h*_ and *PC*_*m*_ it is important to collect more accurate data on line spacing and how it differs between farms.

Valderrama et al. (2013, 2015) also stress the difficulties of estimating labour costs in farming systems with a significant share of unpaid family labour. In that case they propose to estimate labour costs as family labour input multiplied by wages of hired labour. To benchmark seaweed cultivation systems, Valderrama calculated labour costs per kg harvested seaweed (US$ kg^-1^ semi-dry seaweed), a sensible approach for comparing productivity of these labour intensive farming system across the world. In BESeM we intentionally kept the model as simple as possible, which means costs were not further split up into labour and non-labour costs. Such an extension of the model would be relatively simple to implement. Also Zuniga-Jara and Marin-Riffo (2016) present more detailed analyses of different categories of production costs and methods for calculating Net Present Value (NPV) that may be considered in future studies - see also our discussion on production costs in the section "Recurring operating costs and investments".

An additional challenge with the economic parameters compared with the biological parameters is that the economic parameters are, more than the biological parameters, subject to changes over time. The COVID-19 pandemic which started early 2020 has led to a collapse of the tourism sector in Indonesia. Part of the people working in tourism have moved out of tourism and into seaweed farming. It has led to an increase in labour availability which has led to a drop in wages and it has led to increase supply of seaweed which in turn has led to a large drop in farmgate prices (Langford et al. 2021). In this study we used farmgate prices and production costs recorded in the study site in 2019. It is unclear how much production costs have dropped in the current situation, which makes it difficult to make current net income estimates. For new studies it is recommendable to make new site specific estimates of the BESeM economic parameters.

## Conclusions

A simple model was presented for simulating production and economics of tropical seaweed cultivation. The model has a limited number of parameters which makes it easily amenable to other seaweeds and other sites. Parameters for *Gracilaria* were estimated for a site in Indonesia and uncertainties in parameters was discussed. A simulation example suggests the model can be used to simulate optimum farm management for maximising gross or net income. Since parameters of the model are still quite uncertain we caution against using outcomes of this paper directly for recommendations for farmers. Instead, we recommend more research: validation and more accurate parameter estimation.

## Supplementary Information

ESM 1 (CSV 666 bytes)

ESM 2 (CSV 18 kb)

ESM 3 (CSV 23 kb)

ESM 4 (CSV 31 kb)

ESM 5 (R 38 kb)

ESM 6 (CSV 869 bytes)

ESM 7 (CSV 12 kb)

## Data Availability

The datasets generated during and/or analysed during the current study are available as supplementary material.

## Footnotes

1 We will use the word “plant” and “farm” as in the more agronomic sense of farming. In strict biological sense the word “plant” is not appropriate, as the seaweed is a macro alga. The word “cutting” instead of “plant” could be used to indicate that a part of each harvested seaweed individual is cut off for being replanted (and the remainder is dried and sold).

## Code availability

The code generated during and/or analysed during the current study are available as supplementary material.
